# KLF7 Blocks MKNK2/HIF‐1 Pathway‐Mediated M1 Microglia Polarization to Ameliorate Ischemic Stroke‐Induced Neurological Injury

**DOI:** 10.1002/brb3.70850

**Published:** 2025-09-09

**Authors:** Ran Wei, Shuang Li, Lei Tang

**Affiliations:** ^1^ Department of Neurology The First Affiliated Hospital of Harbin Medical University Harbin Heilongjiang P. R. China

**Keywords:** HIF‐1 pathway, Ischemic stroke, KLF7, MKNK2, microglia

## Abstract

**Background:**

Ischemic stroke (IS) is a common neurological disease with a significant financial burden but lacks effective drugs. This study sought to explore the mechanisms underlying MAP kinase‐interacting serine/threonine‐protein kinase 2 (MKNK2), a gene enriched in the hypoxia‐inducible factor‐1 (HIF‐1) signaling, in IS‐related neurological injury.

**Methods:**

Middle cerebral artery occlusion/reperfusion (MCAO/R) and oxygen‐glucose deprivation/reoxygenation (OGD/R) models were used in vivo and in vitro. Rats were infected with lentiviral vectors harboring knockdown or overexpression of the target genes, followed by MCAO/R to conduct 2,3,5‐triphenyltetrazolium chloride, HE, Fluoro‐Jade C, and TUNEL, enzyme‐linked immunosorbent assay, immunohistochemistry, and neurological deficits assessment. The regulation of Krueppel‐like factor 7 (KLF7) on MKNK2 was analyzed by ChIP and dual‐luciferase assays. The effects of the KLF7/MKNK2/HIF‐1 axis on the M1 or M2 polarization of rat microglia were demonstrated by the transfection of knockdown or overexpression plasmids into the cells.

**Results:**

MCAO/R‐treated rat brain tissues and OGD/R‐treated rat microglia showed MKNK2 upregulation along with activation of the HIF‐1 signaling, whereas KLF7 expression was downregulated. Knockdown of MKNK2 inhibited the HIF‐1 signaling and M1 microglia polarization, whereas it promoted M2 polarization. KLF7 repressed the MKNK2 transcription, thereby achieving the same effect as the knockdown of MKNK2 in vitro, which was reversed by combined overexpression of MKNK2. Knockdown of MKNK2 or overexpression of KLF7 ameliorated MCAO/R‐induced brain damage and neurological injury in rats. MKNK2 overexpression reversed the alleviating effect of KLF7 overexpression on pathological brain injury in rats.

**Conclusion:**

Significant downregulation of KLF7 expression after IS exacerbated pathological brain damage through the MKNK2‐mediated HIF‐1 pathway.

## Graphical abstract

Schematic diagram. Low KLF7 expression after IS further impairs transcriptional repression of MKNK2, which exacerbates MCAO/R‐induced pathological injury in mice by activating the HIF‐1 pathway‐mediated pro‐inflammatory M1 polarization of microglia.

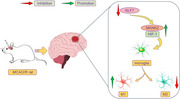

**Abbreviations**
ECA: external carotid artery
ELISA: enzyme‐linked immunosorbent assay
HE: hematoxylin‐eosin
HIF‐1: hypoxia‐inducible factor‐1
IS: ischemic stroke
KLF: Krueppel‐like factor
MCAO/R: middle cerebral artery occlusion/reperfusion
MKNK2: MAP kinase‐interacting serine/threonine‐protein kinase 2
OGD/R: oxygen‐glucose deprivation/reoxygenation
TTC: 2,3,5‐triphenyltetrazolium chloride

## 1 Introduction

Stroke is the leading cause of disabilities and cognitive deficits, responsible for 5.2% of all mortalities worldwide, and transient or permanent occlusion of cerebral vessels leads to ischemic strokes (IS), which constitute the majority of stroke cases (Zhao et al. 2022). Intravenous thrombolysis remains the standard treatment of acute IS, while nurses continue to play a pivotal role in the care of patients with IS through the coordination of care (Rodgers et al. 2021). There is still a substantial need for the development of therapeutic agents to protect the brain from damage before and during recanalization, extend the therapeutic time window, and improve functional outcomes (Paul and Candelario‐Jalil 2021). Microglia, serving as resident innate immune cells in the central nervous system, undergo pro‐inflammatory or anti‐inflammatory phenotypes in response to the microenvironmental changes following IS (Planas 2024; Qiu et al. 2021). Therefore, it is valuable to identify the mechanisms underlying microglia polarization, which may provide a promising treatment strategy for neuronal damage after IS.

Hypoxia‐inducible factor‐1α (HIF‐1α) is a sensitive regulator of oxygen homeostasis, and its expression is rapidly induced after hypoxia/ischemia, thereby playing an extensive role in the pathophysiology of IS by regulating neuronal survival and microglia‐mediated neuroinflammation (He et al. 2021; Vatte and Ugale 2023). Mitogen‐activated protein kinase‐interacting serine/threonine‐protein kinases (MKNK, also known as MNK) represent a class of enzymes that are activated by an extracellular signal‐regulated kinase or p38 MAPK and control the production of inflammatory chemokines, and regulate cell proliferation and survival (Xu et al. 2022). This family has been widely investigated in the initiation and progression of malignancies (Mazewski and Platanias 2023; Prabhu et al. 2020), while their role in IS has not been revealed. In our preliminary bioinformatics prediction, MAP kinase‐interacting serine/threonine‐protein kinase 2 (MKNK2) has been revealed as one of the significantly differentially expressed genes in microglia following IS, which was enriched in the HIF‐1 pathway. Interestingly, the expression of its counterpart MKNK1 has been reported to be regulated by a transcription factor, friend leukemia integration 1, in leukemic cells (Wang et al. 2020). Therefore, we anticipated that the expression of MKNK2 was regulated by a transcription factor in IS as well. The Kruppel‐like factor (KLF) family proteins, which have 18 members, are transcription factors that can regulate the activation and suppression of transcription, and KLF4, KLF7, KLF8, and KLF9 were found to be related to the nervous system (Yuce and Ozkan 2024). KLF7 is highly expressed in three periods during the development of the central nervous system in mice, and upregulation of KLF7 has been proposed as an effective way to treat nerve injury (Mao et al. 2023). However, whether KLF7 can alleviate neuroinflammation in IS has not been studied. In this study, we investigated whether KLF7 induced anti‐inflammatory properties by controlling microglia polarization in vivo and in vitro and whether the underlying mechanism was related to the MKNK2/HIF1‐α pathway.

## 2 Materials and Methods

### 2.1 Differential Expression Analysis

The GSE148350 dataset on the public GEO database (https://www.ncbi.nlm.nih.gov/geo/query/acc.cgi?acc=GSE148350) was analyzed using the GEO2R method on the (RaGene‐1_0‐st) Affymetrix Rat Gene 1.0 ST Array (transcript [gene] version) array. Microglia isolated from rats 3–14 days after IS (*n* = 24) were defined as the IS group, and six microglia isolated from sham‐operated rats were defined as the sham group. Benjamini–Hochberg (false discovery rate) was applied for *p*‐value correction. Significance level cut‐off = 0.01 was set as the screening threshold. Transcriptome sequencing results are presented as a volcano plot.

### 2.2 Animal Preparation

Adult male CD (SD) IGS rats (101) (250–300 g) at 8 weeks of age were purchased from Beijing Vital River Laboratory Animal Technology Co., Ltd. (Beijing, China) and housed on a 12‐h light/dark cycle. The procedures received approval from the Animal Care and Use Committee of the First Affiliated Hospital of Harbin Medical University. They were conducted following the Guide for the Care and Use of Laboratory Animals (revised 1996).

### 2.3 Intracerebral Microinjection

SD rats were anesthetized with 75 mg/kg ketamine and 5 mg/kg valium (i.p.) and placed in a stereotaxic frame. Lentiviruses harboring short hairpin RNA (sh)‐MKNK2, overexpression (oe)‐KLF7, and their control lentiviruses (sh‐Scramble or oe‐NC) purchased from Origene (Beijing, China) with titers ranging from 2 × 10^8^ to 8 × 10^8^ CFU/mL were microinjected into the posterior fontanel position of the right ventricle at the following coordinates from bregma: −0.9 mm anterior, ± 1.5 mm lateral, and −3.6 mm deep. Two microliters of lentivirus were injected at a rate of 1 µL/min, and the needle was retained in place for 5 min following the injection (Wang et al. 2020).

### 2.4 Establishment of Middle Cerebral Artery Occlusion/Reperfusion (MCAO/R)

The MCAO rat modeling was initiated 12 days after the lentiviral injection. SD rats were anesthetized as aforementioned. A thermostatic blanket was used to maintain the rectal temperature of the rats within 37.0 ± 0.5°C. The rats were placed supine, and a midline incision was made in the neck to expose the right external carotid artery (ECA), common carotid artery, and internal carotid artery (ICA). An incision between the two ligation points of the ECA was made. A 4‐0 silicone‐coated monofilament suture was inserted through the ECA into the right ICA and advanced (18–22 mm) until resistance was felt. After 2 h of occlusion, the sutures were removed from the ECA to achieve reperfusion (Zhou et al. 2021). Sham‐operated rats underwent the same surgery but without occlusion. Rats were euthanized by intraperitoneal injection of 200 mg/kg sodium pentobarbital 24 h after MCAO/R surgery, and brains were rapidly collected for subsequent experiments.

### 2.5 Neurological Deficiency Assessment

Neurobehavioral dysfunction was assessed by using the following criteria in rats 24 h after MCAO/R. Neurological scores follow a five‐point scale: 0: *no neurological deficits*; 1: *inability to extend the right forelimb*; 2: circling to the contralateral side; 3: bending over to the injured side; 4: lack of spontaneous motor activity and presence of a low level of consciousness (Longa et al. 1989).

### 2.6 2,3,5‐Triphenyltetrazolium Chloride (TTC)

After 24 h of MCAO in rats, the brains were rapidly removed from rats at 4°C. The brains were then cut into coronal slices of 2 mm, stained with PBS containing 2% TTC (T8877, Merck Millipore, Darmstadt, Germany) solution for 20 min at room temperature, and fixed in 4% paraformaldehyde at 4°C for 24 h. The volumes of contralateral and non‐infarcted tissue of the ipsilateral hemispheres were measured with ImageJ software (NIH). The following formula was used: Infarct area (%) = (infarct area)/(contralateral hemisphere area + ipsilateral hemisphere area) × 100% (Kim et al. 2022).

### 2.7 Dual‐Labeling Immunofluorescence Staining

The brains of rats were isolated, fixed, dehydrated, and cut into 10 µm sections. The sections were dewaxed, hydrated, and treated with 3% H_2_O_2_ for 10 min at room temperature, followed by antigen retrieval. The sections were treated with goat serum (R37624, Invitrogen Inc., Carlsbad, CA, USA) for 40 min at 37°C and incubated with anti‐iba1 (1:100, ab283319, Abcam, Cambridge, UK), anti‐GFAP (1:50,14‐9892‐82, Invitrogen), anti‐NeuN (1:500, MA5‐33103, Invitrogen), anti‐Olig2 (1:20, 615952, BioLegend, San Diego, CA, USA), and anti‐MKNK2 (1:100, MBS9463045, MyBioSource, Inc., San Diego, CA, USA) overnight at 4°C. The sections were incubated with goat anti‐rabbit IgG H&L (Alexa Fluor 488) (1:200, ab150077, Abcam), goat anti‐mouse IgG H&L (Alexa Fluor 647) (1:200, ab150115, Abcam), or goat anti‐rat IgG Alexa Fluor 647 (1:500, A‐21247, Invitrogen) for 1 h at 25°C. Images were observed with a fluorescence microscope.

### 2.8 Hematoxylin‐Eosin (HE) Staining

After being dewaxed and hydrated using the above routine procedure, rat brain sections were stained using hematoxylin (C0105S, Beyotime Biotechnology Co., Ltd., Shanghai, China) for 5 min, treated with 1% hydrochloric acid ethanol for 25 s, and 1% ammonia for 30 s, stained with eosin for 5 min, and finally dehydrated with increasing concentrations of ethanol and permeabilized with xylene. The cell death in the cerebral cortex was viewed under a light microscope, and positive cells were counted.

### 2.9 Fluoro‐Jade C (FJC) Staining

FJC labeling staining was performed using the FJC Ready‐to‐Dilute Staining Kit for identifying degenerating neurons (TR‐100‐FJT, AmyJet Scientific Inc., Wuhan, Hubei, China). Briefly, rat brain tissues were fixed with 4% paraformaldehyde and made into coronal sections. Brain sections were first immersed in 80% ethanol containing 1% NaOH for 5 min, followed by rinsing in 70% concentration ethanol and distilled water for 2 min each, and incubated for 10 min using 0.06% KMnO_4_ solution. Brain sections were transferred to 0.0001% FJC solution for 10 min incubation at room temperature in the dark, air‐dried, and treated with xylene. After neutral gum sealing, the brain sections were photographed using a fluorescence microscope. FJC‐positive cells were counted by ImageJ software.

### 2.10 TUNEL Assay

Apoptosis was evaluated using the In Situ Cell Death Detection Kit (C10617, Invitrogen). Briefly, brain sections were incubated with the TUNEL reaction mixture at room temperature and washed with PBS before staining the sections with 4′,6‐diamidino‐2‐phenylindole (DAPI). Stained tissues were observed under a fluorescence microscope and quantified using ImageJ software.

### 2.11 Immunohistochemistry

Paraffin‐embedded sections were baked in an oven at 60°C for 20 min, deparaffinized in each xylene (I, II, and III) for 5 min, hydrated with anhydrous ethanol (I and II), 90% ethanol (I and II), 80% ethanol (I and II), and 70% ethanol (I and II) for 2 min each, washed with water, and transferred to distilled water for 2 min and washed with PBS for 5 min. The sections were immunostained with PBS‐diluted anti‐CD16 (1:100, NBP2‐42228, Novus Biological Inc., Littleton, CO, USA) overnight at 4°C and with diluted goat anti‐mouse IgG (H+L) secondary antibody (1:500, ab205719, Abcam) for 30 min at 37°C. The sections were developed with 200 µL of 3,3′‐diaminobenzidine tetrahydrochloride (34065, Thermo Fisher Scientific Inc., Waltham, MA, USA) working solution, followed by counterstaining with 100 µL hematoxylin staining solution for 10 min, dehydration, and sealing. The sections were visualized under a microscope. The immunohistochemical staining was quantified by the positive staining.

### 2.12 Cell Culture and Plasmid Transfection

Rat microglia (CP‐R110, Procell, Wuhan, Hubei, China) were cultured in DMEM containing 10% FBS. MKNK2 siRNA and control siRNA (si‐NC); overexpression plasmids for MKNK2 and KLF7 and control plasmid (oe‐NC) were designed and obtained from Origene. The above siRNA or overexpression plasmids were transfected into cells by EndoFectin MAX transfection reagent (EF013, Guangzhou iGene Biotechnology Co., Ltd., Guangzhou, Guangdong, China) according to the manufacturer's instructions. To verify the transfection efficiency of microglia with plasmids, plasmids with green fluorescent protein (GFP) were used for transfection, which was observed using fluorescence microscopy 48 h after transfection. The transfection efficacy was analyzed by RT‐qPCR and western blot analysis.

Within 48 h after transfection, the transfected cells were treated with 10 µM Fenbendazole‐d_3_ (HY‐B0413S, MedChemExpress, Monmouth Junction, NJ, USA) for 4 h to activate HIF‐1α signaling, and the control group was treated with the solvent DMSO (Aleyasin et al. 2015).

For the development of the oxygen‐glucose deprivation/reoxygenation (OGD/R) cell model, transfected or activator‐treated cells were maintained for 2 h in glucose‐free medium in a humidified incubator containing 95% N_2_ and 5% CO_2_. The medium was then replaced with DMEM and returned to the normal incubator to reoxygenate for 24 h. The cells treated with a normal medium under normoxic conditions were used as the control group (Con) (Y. Li et al. 2023).

### 2.13 RT‐qPCR

Rat brain tissues were taken from SD rats or cells with different treatments. TRIzol reagent (15596026CN, Thermo Fisher) was used to extract total RNA from animal tissues or cells, and RNA was reverse transcribed into cDNA using the LunaScript RT Master Mix Kit (E3025S, New England Biolabs, Ipswich, MA, USA). Real‐time PCR was performed using the 7500 Fast Real‐Time PCR (4351106, Thermo Fisher) using Luna Universal qPCR Master Mix (M3003V, New England Biolabs). The relative levels were calculated with the 2^−ΔΔCT^ method using GAPDH as the internal standard control. The primers are listed in Table 1.

**TABLE 1:** RT‐qPCR primers.

Gene name (species)	Forward primer (5′–3′)	Reverse primer (5’‐3’)
MKNK2 (Rattus norvegicus)	CGCTCTCCTCGCTACTTCTG	AGGGCCATGGAGAAAATGGG
KLF7 (Rattus norvegicus)	TTACTCCCGTGAAGTCCCCT	AGTCCAAGTCCTCGCCAAAG
CD16 (Rattus norvegicus)	TCCGTGGCAGTCTATGAGGA	CAGATGGTGAGGTCGCAAGT
iNOS (Rattus norvegicus)	AGTCAACTACAAGCCCCACG	GCAGCTTGTCCAGGGATTCT
Arg‐1 (Rattus norvegicus)	ATGTGCCCTCTGTCTTTTAGGG	GCCGATGTACACGATGTCCT
CD206 (Rattus norvegicus)	TCAGAGCAAACTGCGTGGAT	CCGCATGCTCATTCTGTTCG
GAPDH (Rattus norvegicus)	GCATCTTCTTGTGCAGTGCC	GATGGTGATGGGTTTCCCGT
HIF‐1α (Rattus norvegicus)	GCAACTAGGAACCCGAACCA	AGATGGGAGCTCACGTTGTG

Abbreviations: GAPDH, glyceraldehyde‐3‐phosphate dehydrogenase; iNOS, inducible nitric oxide synthase; KLF7, Krueppel‐like factor 7; MKNK2, MAP kinase‐interacting serine/threonine‐protein kinase 2.

### 2.14 Western Blot Analysis

The differently treated cells were incubated in M‐PER mammalian protein extraction reagent (78501, Thermo Scientific) for 15 min and collected by centrifugation at 12,000 *g* at 4°C for 30 min. The total protein concentration in the lysates was measured using the BCA Protein Assay Kit (CW0014S, CW Biotechnology Co., Ltd., Beijing, China). Equal amounts of protein extracts were separated using SDS‐PAGE and transferred to a PVDF membrane. The membrane was blocked with 5% skim milk powder diluted in TBST for 1 h and incubated with primary antibodies at 4°C overnight and secondary antibodies, goat anti‐mouse IgG H&L (HRP) (1:10000, ab205719, Abcam) or goat anti‐rabbit IgG, HRP (1:10000, 31460, Invitrogen) at room temperature for 1 h. The following antibodies were used: anti‐MKNK2 (1:500, H00002872‐M07, Novus Biological), anti‐HIF‐1α (1:500, NB100‐105SS, Novus Biological), anti‐KLF7 (1:3000, MBS9129225, MyBioSource), and anti‐GAPDH (1:5000, ab8245, Abcam). Proteins were visualized using the Ultrasensitive ECL Detection Kit (PK10003, ProteinTech Group, Chicago, IL, USA), and blots were quantified using ImageJ software.

### 2.15 Immunocytochemistry

Rat microglia were fixed with 4% paraformaldehyde for 10 min at room temperature, permeabilized with 0.1% Triton X‐100 for 5 min, and sealed with PBS solution containing 5% goat serum for 4 h. Briefly, the cells were labeled with antibodies against CD16 (1:200, NBP2‐42228, Novus Biological) and Arg‐1 (1:250, NBP1‐32731, Novus Biologicals) overnight at 4°C. They were then incubated with the secondary antibodies, goat anti‐rabbit IgG H&L (Alexa Fluor 488) (1:500, ab150077, Abcam) or goat anti‐mouse IgG H&L (Alexa Fluor 647) (1:500, ab150115, Abcam) for 1 h at room temperature, and with DAPI for nuclear staining. Finally, images were acquired using fluorescence microscopy and analyzed for relative fluorescence intensity using Image J software.

### 2.16 Enzyme‑Linked Immunosorbent Assay (ELISA)

The levels of IL‐1β (BMS630, Thermo Fisher), IL‐6 (BMS625, Thermo Fisher), and IL‐10 (BMS629, Thermo Fisher) in microglia and rat brain tissues were measured by ELISA according to the instructions of the ELISA kits. The samples (100 µL) were incubated at 37°C for 90 min and treated with Biotinylated Antibody Working Solution (Concentrated Biotinylated Detection Ab: Biotinylated Detection Ab: Diluent = 1:100) for 60 min at 37°C. After that, the samples were incubated with a coupler (Concentrated HRP Coupler: HRP Conjugate Diluent = 1:100) for 30 min and with substrate reagent (TMB) for 15 min (both at 37°C). The OD of each well was measured at 450 nm using a microplate reader.

### 2.17 Chromatin Immunoprecipitation (ChIP) Assay

Microglia were fixed in 4% paraformaldehyde. DNA was sheared by sonication and added to the ChIP dilution buffer. A proportion of the sample (5 µL) was used as INPUT. The precleared chromatin solution was incubated overnight at 4°C with anti‐KLF7 (1:25, MBS9129225, MyBioSource) and rabbit IgG isotype control (1:100, ab171870, Abcam). DNA was purified from the complex, captured, washed, eluted, and de‐crosslinked. qPCR was performed on the precipitated DNA.

### 2.18 Luciferase Reporter Assays

The luciferase reporter gene assay was performed according to the manufacturer's protocol (Promega Corporation, Madison, WI, USA). A pGL3‐based luciferase reporter vector containing the MKNK2 promoter and the Renilla luciferase plasmid was transfected into the cells using Lipofectamine 3000 (Invitrogen). After 48 h post‐transfection, luciferase activity was assayed by a dual luciferase reporter assay system, and firefly luciferase activity was normalized to Renilla activity.

### 2.19 Statistical Analysis

All data from individual experiments were shown as mean ± standard error of the mean (SEM). In vitro experiments were repeated three times to confirm the results. All statistical analyses were performed with GraphPad Prism version 10.4.2 (GraphPad Software, Inc., San Diego, CA). Statistical comparisons were assessed by unpaired *t*‐test, one‐way ANOVA, or two‐way ANOVA with Tukey's test for multiple comparisons. Statistical significance was set at *p* < 0.05.

## 3 Results

### 3.1 MKNK2 and HIF‐1α Expression are Upregulated in Microglia During IS

**FIGURE 1:**
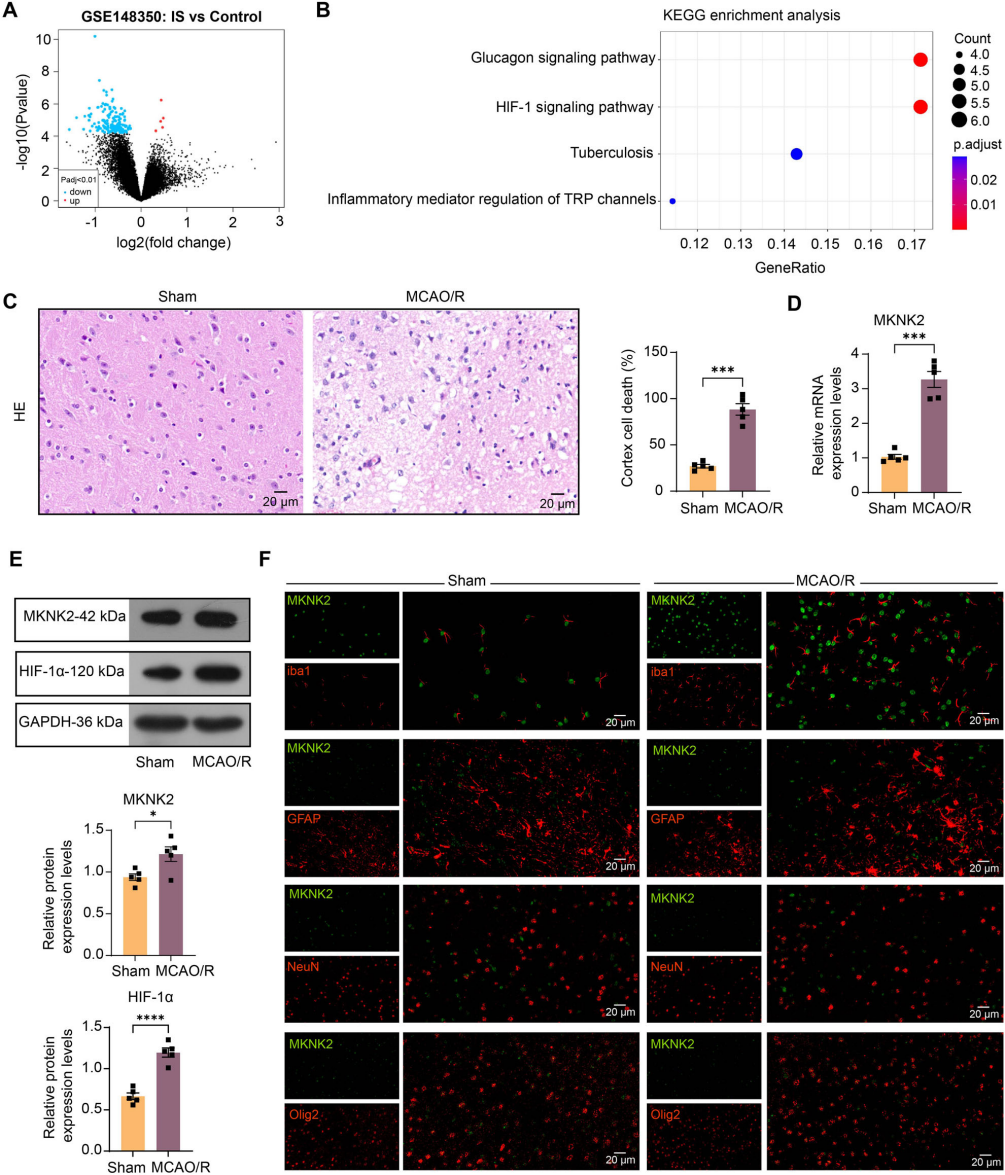
MAP kinase‐interacting serine/threonine‐protein kinase 2 (MKNK2) and hypoxia‐inducible factor‐1α (HIF‐1α) expression are significantly upregulated in microglia after IS. (A) Differentially expressed genes between the microglia from IS rat brains and the sham‐operated rats in the GSE148350 dataset were analyzed using adj. *p* < 0.01 as a threshold. (B) KEGG pathway enrichment analysis of 79 intersecting genes. (C) Histopathology of the cortex of the sham and middle cerebral artery occlusion/reperfusion (MCAO/R)‐operated rats assessed by HE staining. (D) The mRNA expression of MKNK2 in brain tissues of sham and MCAO/R‐operated rats was analyzed using RT‐qPCR. (E) Protein levels of MKNK2 and HIF‐1α in brain tissues of sham and MCAO/R‐operated rats were analyzed by Western blot analysis. (F) MKNK2 expression (green) in astrocytes (GFAP^+^), neurons (NeuN^+^), oligodendrocytes (Olig2^+^), and microglia (iba1^+^) in sham and MCAO/R‐operated rats was analyzed by dual‐labeling immunofluorescence staining. Values are mean ± SEM, *p < 0.05, ***p < 0.001, and ****p < 0.0001. *n* = 5 per group. *p*‐values were calculated using an unpaired *t*‐test.

We analyzed the microglia transcriptome in the GSE148350 dataset in the GEO database (Figure 1A) (adj. *p* < 0.01). KEGG pathway enrichment analysis (Figure 1B) was then performed on significantly differentially expressed genes (*n* = 79, Supporting Information) in Hiplot Pro (https://hiplot.com.cn/). We found that significant enrichment of the HIF‐1 pathway, which has been identified as an important regulator in IS (Vatte and Ugale 2023). Among the six genes enriched in this pathway, EP300 (Barrera‐Vazquez et al. 2022), CAMK2G, and CAMK2D (Ye et al. 2019) have been implicated in IS. MKNK2, which is shown by the KEGG map to activate the HIF‐1 pathway (Figure S1), piqued our attention.

We developed an IS rat model via MCAO/R surgery, followed by HE staining. The cortical histopathology was normal in the sham group, and most of the cells were disorganized in the MCAO/R group with a large number of necrotic cells (Figure 1C), which proved that the modeling was successful. RT‐qPCR was performed on brain tissue homogenates of animals at 24 h after reperfusion, and the mRNA expression of MKNK2 in the brain homogenates of rats induced by MCAO/R was higher than that of sham‐operated rats (Figure 1D). Moreover, the results of Western blot analysis showed that the protein expression of both MKNK2 and HIF‐1α was enhanced following MCAO/R surgery (Figure 1E). To verify the distribution of MKNK2 in different cell types of MCAO/R rat brain tissues, we labeled MKNK2 with different markers, including astrocytes (GFAP), neurons (NeuN), oligodendrocytes (Olig2), and microglia (iba1), using immunofluorescence. In the brain tissues from sham‐operated rats, MKNK2 was expressed in astrocytes, neurons, oligodendrocytes, and microglia. More importantly, only microglia showed the most prominent upregulation of MKNK2 expression after MACO/R in rats (Figure 1F).

### 3.2 Overactivation of HIF‐1α Signaling Reverses M1 Microglia Polarization Blocked by si‐MKNK2

**FIGURE 2:**
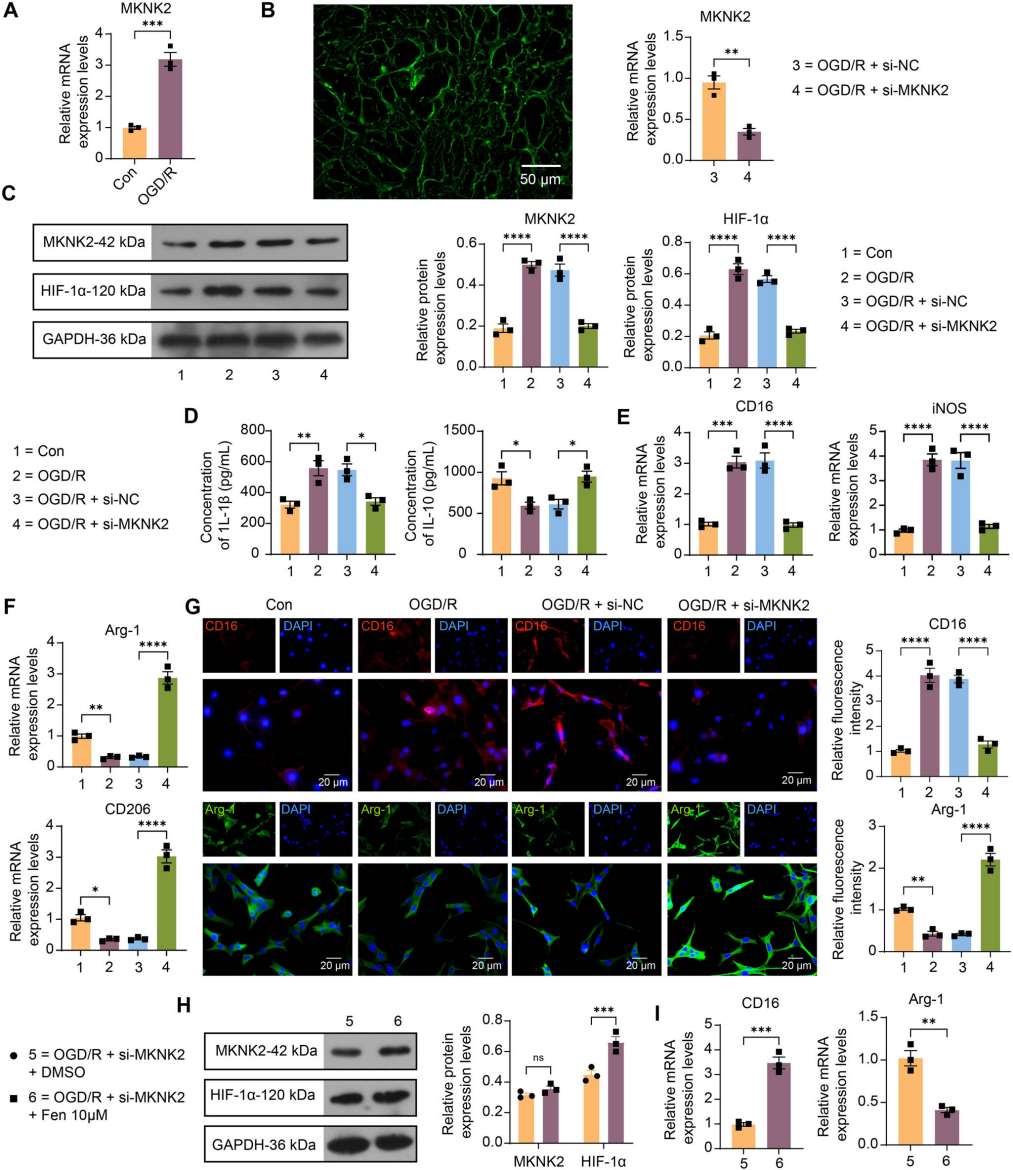
MAP kinase‐interacting serine/threonine‐protein kinase 2/hypoxia‐inducible factor‐1α (MKNK2/HIF‐1α) axis is critical for M1 polarization in rat microglia exposed to oxygen‐glucose deprivation/reoxygenation (OGD/R). (A) mRNA expression of MKNK2 in microglia exposed to OGD/R or not was assessed by RT‐qPCR. (B) Plasmid transfection efficiency was observed using fluorescence microscopy, and mRNA expression of MKNK2 in microglia after knockdown of MKNK2 was assessed by RT‐qPCR. (C) The protein expression of MKNK2 and HIF‐1α in cells with different treatments was assessed by Western blot assays. (D) Detection of inflammatory factors IL‐1β and IL‐10 secreted by microglia by enzyme‐linked immunosorbent assay (ELISA). (E) Expression of genes related to M1 phenotype (CD16 and iNOS) in microglia after OGD/R and knockdown of MKNK2 detected by RT‐qPCR. (F) Expression of genes related to M2 phenotype (Arg‐1 and CD206) in microglia after OGD/R and knockdown of MKNK2 was detected by RT‐qPCR. (G) The relative fluorescence intensity of M1 phenotype (CD16) or M2 phenotype (Arg‐1) in rat microglia after OGD/R and knockdown of MKNK2 was detected by immunocytochemistry. (H) Protein expression of MKNK2 and HIF‐1α in OGD/R‐induced cells treated with knockdown of MKNK2 in combination with the HIF‐1α pathway activator, Fenbendazole‐d_3_, was analyzed by Western blot analysis. (I) Expression of CD16 and Arg‐1 in OGD/R‐induced cells treated with knockdown of MKNK2 in combination with the HIF‐1α pathway activator, Fenbendazole‐d_3_, detected by RT‐qPCR. Values are mean ± SEM, *p <  0.05, **p <  0.01, ***p <  0.001, and ****p <  0.0001. *n* = 3. *p*‐values were calculated using an unpaired *t*‐test or ANOVA.

We subjected rat microglia to an OGD/R to mimic the in vitro conditions of IS or transfected with si‐NC or si‐MKNK2 before OGD/R modeling. As analyzed by RT‐qPCR, the mRNA expression of MKNK2 in cells after OGD/R was found to be significantly higher than that in cells exposed to normal conditions (Figure 2A). After transfection of the knockdown MKNK2 plasmid, it was first observed using fluorescence microscopy that the plasmid transfection efficiency was good, and the downregulation of MKNK2 by transfection was verified using RT‐qPCR (Figure 2B). Western blot analysis showed that the protein expression of MKNK2 and HIF‐1α was significantly upregulated in cells after OGD/R, and the expression of MKNK2 and HIF‐1α was significantly reduced after si‐MKNK2 intervention (Figure 2C). The levels of inflammatory factors IL‐1β and IL‐10 secreted by microglia under different polarizations were detected by ELISA. As expected, OGD/R promoted microglia M1 polarization and inhibited M2 polarization, and the knockdown of MKNK2 reversed this trend (Figure 2D). In addition, the mRNA expression of CD16 and iNOS induced by OGD/R was reduced by si‐MKNK2 (Figure 2E). By contrast, the suppressed Arg‐1 and CD206 expression was restored by si‐MKNK2 (Figure 2F). By immunocytochemical analysis, it was found that OGD/R treatment significantly enhanced the fluorescence intensity of CD16 but attenuated that of Arg‐1 in rat microglia, which was negated by combined knockdown of MKNK2 (Figure 2G).

Subsequently, we treated the cells transfected with si‐MKNK2 further with 10 µM HIF‐1α pathway activator fenbendazole‐d_3_ or control DMSO for 4 h. Activation of the HIF‐1α pathway using fenbendazole‐d_3_ was found to reverse the inhibition of HIF‐1α protein expression by si‐MKNK2 but did not affect the protein level of MKNK2 by Western blot assay (Figure 2H). As revealed by RT‐qPCR analysis, fenbendazole‐d_3_ intervention elevated CD16 expression and suppressed Arg‐1 expression in cells after OGD/R exposure (Figure 2I).

### 3.3 MKNK2 Inhibition Significantly Attenuates Brain Injury and Neuronal Damage in MCAO/R Rats

**FIGURE 3:**
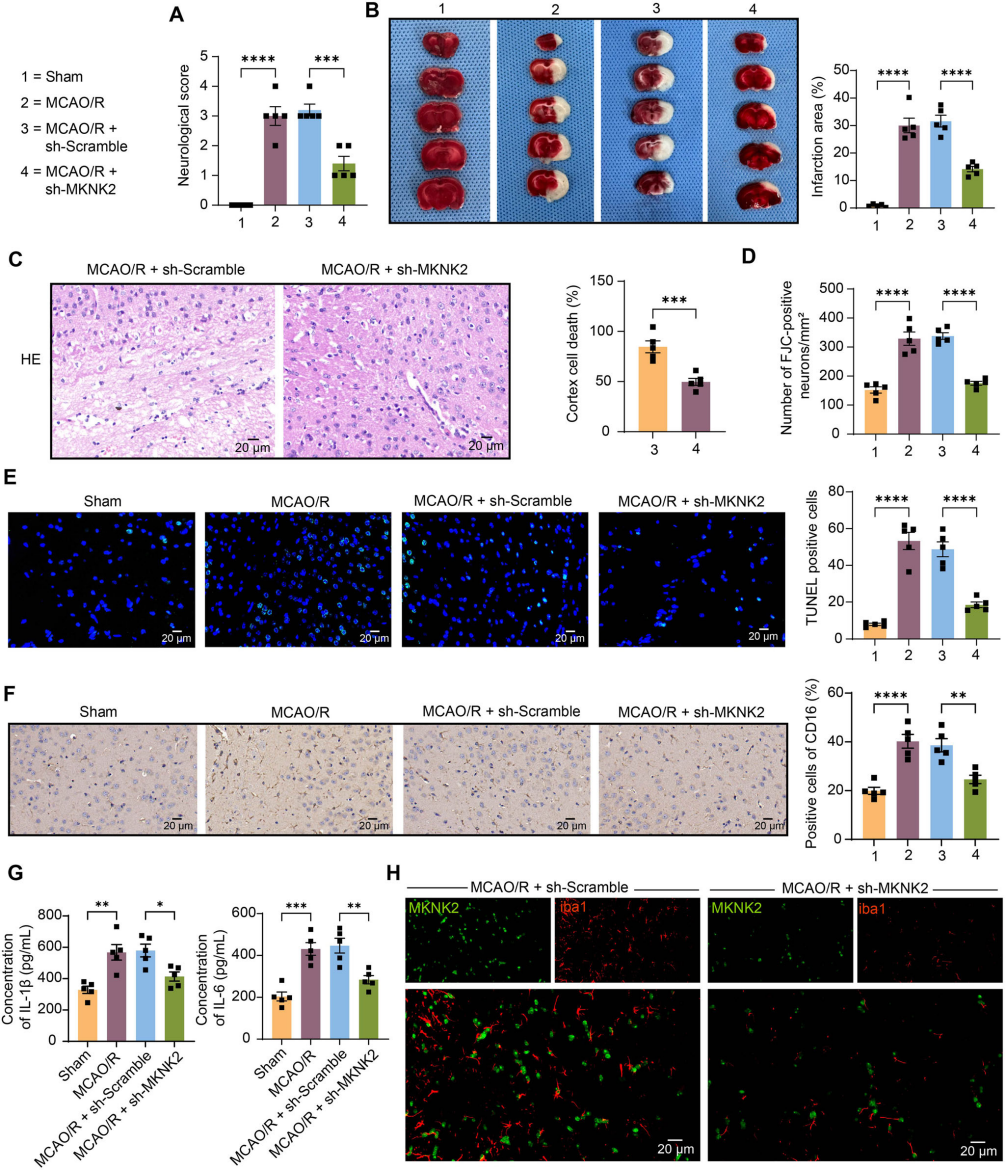
Downregulation of MAP kinase‐interacting serine/threonine‐protein kinase 2 (MKNK2) expression significantly alleviates brain and neuronal injury in rats induced with middle cerebral artery occlusion/reperfusion (MCAO/R). (A) The neurological score of rats was assessed in MCAO/R‐induced rats with MKNK2 knockdown or not. (B) The area of cerebral infarction between the sham‐operated group, the MCAO/R rats, and the lentivirus‐infected MCAO/R rats was analyzed by 2,3,5‐triphenyltetrazolium chloride (TTC) staining. (C) HE staining analysis of brain damage in rats with sh‐MKNK2 induced by MCAO/R. (D) Neuronal degeneration in MCAO/R rats with downregulated MKNK2 expression was analyzed using FJC staining. (E) Detection of apoptosis in sh‐MKNK2‐treated MCAO/R rats by TUNEL. (F) Detection of CD16 in brain tissues of sh‐MKNK2‐treated MCAO/R rats by immunohistochemistry. (G) The content of inflammatory markers (IL‐6 and IL‐1β) secreted by microglia in sh‐MKNK2‐treated MCAO/R rats was analyzed using enzyme‐linked immunosorbent assay (ELISA). (H) Dual‐labeling immunofluorescence analysis of MKNK2 expression (green) in rat brain microglia, iba1 (red). Values are mean ± SEM, *p <  0.05, **p <  0.01, ***p <  0.001, and ****p <  0.0001. *n* = 5 per group. *p*‐values were calculated using an unpaired *t*‐test or ANOVA.

To examine the involvement of MKNK2 in vivo, we delivered lentiviruses harboring sh‐MKNK2 or sh‐Scramble into the brains of rats, followed by the MCAO/R procedure. Neurobehavioral dysfunction was assessed in rats 24 h after MCAO/R. It was found that the neurological deficits of rats were partially recovered by sh‐MKNK2 (Figure 3A). We used TTC staining to analyze coronal brain sections from sham‐operated, MCAO/R, and sh‐MKNK2‐treated MCAO/R rats 24 h after MCAO/R. The results showed that the inhibition of MKNK2 expression significantly reduced the volume of infarctions caused by MCAO/R (Figure 3B). Thereafter, we also performed HE staining on rat coronal brain sections. Neuronal cells in the MCAO/R + sh‐Scramble group showed pustular lesions with proliferative nuclei, whereas silencing of MKNK resulted in more intact nuclei, a significant reduction in pustular lesions, and a significant alleviation of brain damage (Figure 3C). We performed FJC staining of neurons in the infarcted area 24 h after MCAO/R. Knockdown of MKNK2 significantly reduced the number of FJC‐positive degenerating neurons (Figure 3D). Consistently, the cell apoptosis induced by MCAO/R in the brain tissues was significantly repressed by sh‐MKNK2 (Figure 3E). We examined the expression of CD16 in the brain tissues of rats by immunohistochemistry and found that the knockdown of MKNK2 contributed to a significant decrease in the positive cells of CD16 in the brain tissues of rats (Figure 3F). ELISA was used to detect inflammatory markers secreted by microglia, and sh‐MKNK2‐treated MCAO/R rats had decreased pro‐inflammatory factors IL‐1β and IL‐6 (Figure 3G). Finally, by dual‐labeling immunofluorescence of iba1 with MKNK2, we observed that MKNK2 levels were significantly downregulated in microglia (iba1^+^) of rat brains after sh‐MKNK2 lentivirus injection (Figure 3H).

### 3.4 KLF7 Represses MKNK2 Expression in Transcription

**FIGURE 4:**
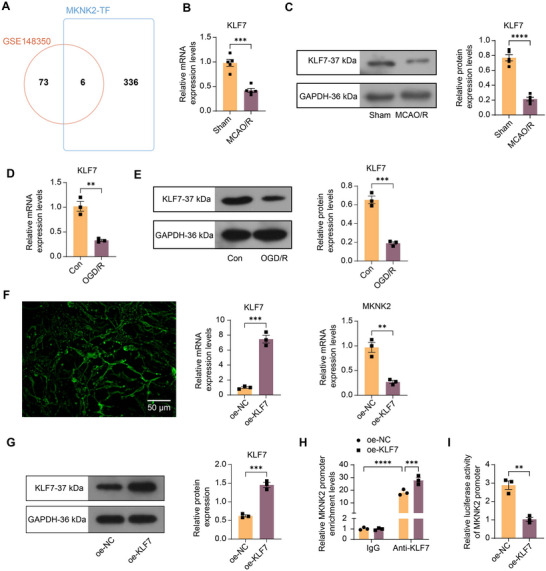
Transcriptional repressor Krueppel‐like factor 7 (KLF7) regulates MAP kinase‐interacting serine/threonine‐protein kinase 2 (MKNK2) expression during IS. (A) The intersection between differentially expressed genes (adj. *p* < 0.01) in the GSE148350 dataset and transcription factors with binding sites at the MKNK2 promoter and enhancer. (B) KLF7 mRNA expression in brain tissues of rats in sham and middle cerebral artery occlusion/reperfusion (MCAO/R) rats was examined using RT‐qPCR. (C) KLF7 protein expression in brain tissues of rats in sham and MCAO/R rats was examined using Western blot analysis. (D) KLF7 mRNA expression in oxygen‐glucose deprivation/reoxygenation (OGD/R)‐induced cells was examined using RT‐qPCR. (E) KLF7 protein expression in OGD/R‐induced cells was examined using Western blot analysis. (F) Plasmid transfection efficiency of oe‐KLF7 transfection was observed using fluorescence microscopy, and mRNA expression of MKNK2 and KLF7 in cells after oe‐KLF7 transfection was examined using RT‐qPCR. (G) The protein expression of KLF7 in cells after oe‐KLF7 transfection was examined using western blot analysis. (H) Detection of KLF7 enrichment level in the MKNK2 promoter region in oe‐KLF7‐treated cells was examined using ChIP. (I) Interaction between KLF7 and the MKNK2 promoter in cells was examined using a dual‐luciferase reporter gene assay. Values are mean ± SEM, **p < 0.01, ***p < 0.001, and ****p < 0.0001. *n* = 3 or 5. *p*‐values were calculated using an unpaired *t*‐test or ANOVA.

To investigate the mechanisms underlying the significantly high expression of MKNK2 during IS, we downloaded transcription factors with binding sites at the MKNK2 promoter and enhancer from Genecards (https://www.genecards.org/) and compared them to the differentially expressed genes (adj. *p* < 0.01) in the GSE148350 dataset on Jvenn. Six intersections—EP300, KLF7, ATF7, RFX3, ZC3H8, and GATAD2B—were found (Figure 4A). We found that the KLF7 was the most significantly under‐expressed one in the GSE148350 dataset (LogFC = −0.8144475), except for EP300, which has been reported in IS as we mentioned above. To this point, we hypothesized that downregulation of the transcriptional repressor KLF7 induces MKNK2 expression, which further promotes the polarization of M1 microglia through activation of HIF‐1 signaling, ultimately exacerbating neurological damage induced by IS.

We first used RT‐qPCR and Western blot analysis to verify whether the expression of KLF7 in the sham‐operated and MCAO/R‐induced rats was consistent with the above prediction results. Unsurprisingly, both the transcription and translation levels of KLF7 in the brains of rats in the MCAO/R group were lower than those of rats in the sham group (Figure 4B,C). To investigate whether M1 polarization of microglia, regulated by MKNK2/HIF‐1α, is mediated by KLF7, we also analyzed the expression of KLF7 in vitro. RT‐qPCR and Western blot assays revealed that compared with the control group, KLF7 mRNA and protein expression were significantly downregulated after OGD/R exposure (Figure 4D,E).

To further explore the potential regulatory relationship between KLF7 and MKNK2, we transfected microglia with oe‐KLF7 vectors (oe‐NC as control) before OGD/R induction. First, microglia stably expressed GFP under fluorescence microscopy (Figure 4F). Moreover, the expression of KLF7 was enhanced after oe‐KLF7 transfection, while that of MKNK2 was reduced (Figure 4F). The upregulation of KLF7 protein was also verified using western blot analysis (Figure 4G). In addition, the enrichment level of KLF7 in the MKNK2 promoter region was significantly upregulated in cells overexpressing KLF7, as revealed by ChIP analysis (Figure 4H). The relative luciferase activity of the MKNK2 promoter luciferase reporter plasmid was significantly reduced after transfection of the KLF7 overexpression plasmid in cells (Figure 4I).

### 3.5 Activation of MKNK2 Induces M1 Polarization of Microglia in the Presence of Oe‐KLF7

**FIGURE 5:**
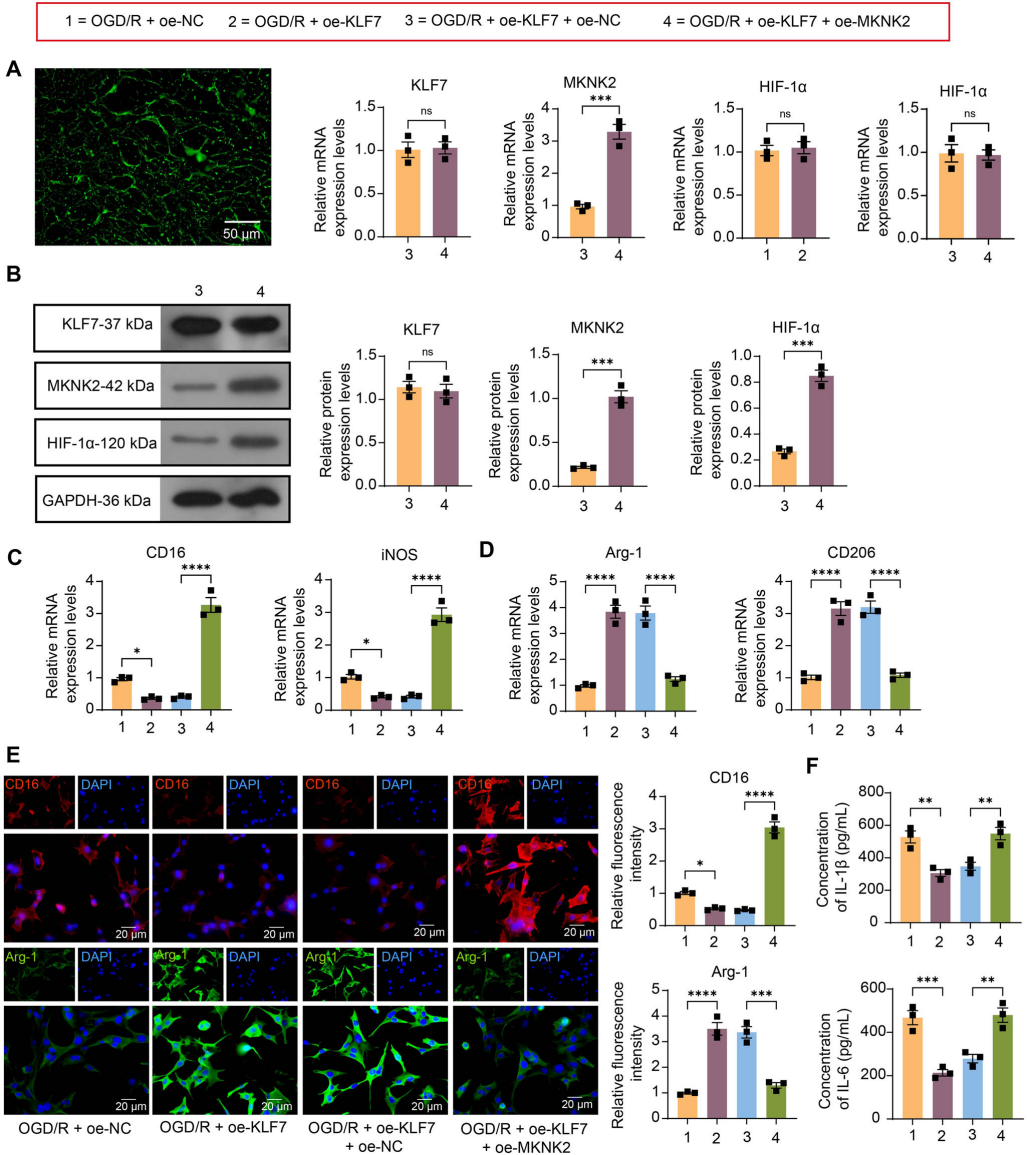
Krueppel‐like factor 7 (KLF7) mediates MAP kinase‐interacting serine/threonine‐protein kinase 2/hypoxia‐inducible factor‐1 (MKNK2/HIF‐1) signaling to promote microglia M1 polarization. (A) Plasmid transfection efficiency was observed using fluorescence microscopy, and mRNA expression of KLF7, MKNK2, and HIF‐1α in cells transfected with oe‐KLF7 alone or combined with oe‐MKNK2 plasmid before oxygen‐glucose deprivation/reoxygenation (OGD/R) induction was examined using RT‐qPCR. (B) The protein expression of KLF7, MKNK2, and HIF‐1α in cells transfected with oe‐KLF7 + oe‐MKNK2 or oe‐NC before OGD/R induction was examined using Western blot. (C) The expression of M1 microglia‐related genes (CD16 and iNOS) in the cells was examined using RT‐qPCR. (D) The expression of M2 microglia‐related genes (CD206 and Arg‐1) in the cells was examined using RT‐qPCR. (E) The relative fluorescence intensity of M1 phenotype (CD16) or M2 phenotype (Arg‐1) in rat microglia was detected by immunocytochemistry. (F) The contents of IL‐1β and IL‐6 in the cells were evaluated using enzyme‐linked immunosorbent assay (ELISA). Values are mean ± SEM, *p < 0.05, **p < 0.01, ***p < 0.001, and ****p < 0.0001. *n* = 3. *p*‐values were calculated using an unpaired *t*‐test or ANOVA.

The microglia were co‐transfected with oe‐KLF7 + oe‐MKNK2 or oe‐KLF7 + oe‐NC, followed by OGD/R. Successful transfection of the above plasmids in microglia was first determined using fluorescence microscopy. As shown in Figure 5A,B, ectopic expression of MKNK2 induced the expression of MKNK2 in the absence of KLF7 expression alteration. We also observed that the protein expression of HIF‐1α was restored following MKNK2 overexpression (Figure 5B). Most importantly, the upregulation of KLF7 expression significantly repressed the expression of genes related to the microglia M1 phenotype, which was enhanced by the reactivation of MKNK2 (Figure 5C). The expression of M2 microglia‐associated genes was, nevertheless, upregulated by the activation of KLF7, and this trend was repressed by the overexpression of MKNK2 (Figure 5D). Subsequent analysis by immunocytochemistry further revealed that overexpression of KLF7 suppressed M1 (CD16) expression but promoted M2 (Arg‐1) expression, and combined overexpression of MKNK2 resulted in upregulation of CD16 expression and downregulation of Arg‐1 (Figure 5E). Cells overexpressing KLF7 were found to have reduced levels of inflammation as analyzed by ELISA, whereas reactivation of MKNK2 resulted in increased secretion of pro‐inflammatory factors IL‐1β and IL‐6 (Figure 5F).

### 3.6 Overexpression of MKNK2 Accentuates Brain Injury and Neuronal Damage in MCAO/R Rats

**FIGURE 6:**
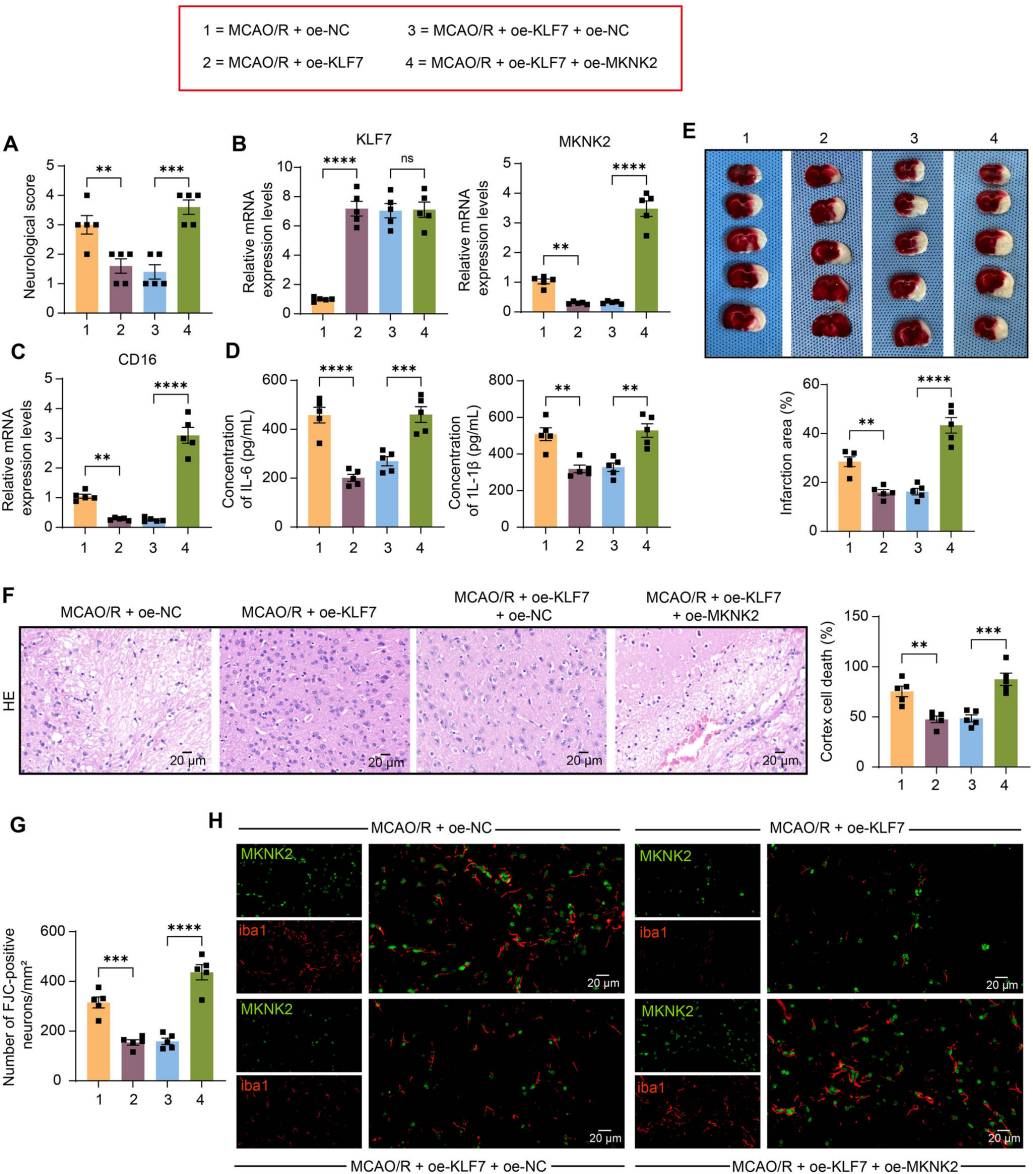
Krueppel‐like factor 7/MAP kinase‐interacting serine/threonine‐protein kinase 2/hypoxia‐inducible factor‐1α (KLF7/MKNK2/HIF‐1α) axis mediates middle cerebral artery occlusion/reperfusion (MCAO/R)‐induced neuronal injury. (A) Assessment of neurobehavioral scores in rats at 24 h postoperatively. (B) The mRNA expression of KLF7 and MKNK2 in brain tissues of oe‐KLF7 combined with oe‐MKNK22 lentivirus‐injected MCAO/R rats was examined using RT‐qPCR. (C) The expression of CD16 in the brain tissues of rats was examined using RT‐qPCR. (D) The concentrations of IL‐1β and IL‐6 in the brain tissues of rats were evaluated using enzyme‐linked immunosorbent assay (ELISA). (E) Detection of cerebral infarction in oe‐KLF7 combined with oe‐MKNK2 lentivirus‐infected MCAO/R rats using 2,3,5‐triphenyltetrazolium chloride (TTC) staining. (F) The degree of brain damage in MCAO/R rats was observed using HE staining. (G) The degeneration of neurons in the brains of the rats was observed using FJC staining. (H) Dual‐labeling immunofluorescence analysis of MKNK2 expression (green) in rat brain microglia, iba1 (red). Values are mean ± SEM, **p < 0.01, ***p < 0.001, and ****p < 0.0001. *n* = 5. *p*‐values were calculated using ANOVA.

Similarly, we performed MCAO/R surgery in rats with oe‐KLF7 alone or in combination with oe‐MKNK2. The neurological deficit in rats was first found to be alleviated by oe‐KLF7, while it was deteriorated by oe‐MKNK2 (Figure 6A). The RT‐qPCR assays on brain tissues of rats with MCAO/R showed that pretreatment of oe‐KLF7 inhibited the expression of MKNK2, whereas overexpression of MKNK2 did not affect the expression of KLF7 (Figure 6B). Analysis by RT‐qPCR revealed that overexpression of KLF7 decreased the expression of CD16 in the brain tissues of MCAO/R rats, which was promoted by the oe‐MKNK2 intervention (Figure 6C). The reduced inflammation, as revealed by the IL‐6 and IL‐1β levels, was enhanced by oe‐MKNK2 (Figure 6D). In addition, TTC showed that overexpression of KLF7 reduced the volume of cerebral infarction in rats compared with the oe‐NC group. In contrast, reactivation of MKNK2 not only offset the alleviation of the cerebral infarction by KLF7 overexpression but also exacerbated this pathology (Figure 6E). Subsequent HE staining revealed that overexpression of MKNK2 reversed the brain damage in rats alleviated by upregulation of KLF7 expression (Figure 6F). Meanwhile, we used FJC staining to analyze neuronal degeneration. Upregulation of KLF7 expression decreased the number of FJC‐positive degenerating neurons, whereas treatment with combined MKNK2 activation increased the number of FJC‐positive degenerating neurons (Figure 6G). Also, dual‐labeling immunofluorescence showed that overexpression of KLF7 downregulated MKNK2 expression in iba1^+^ microglia, which was weakened by combined overexpression of MKNK2 (Figure 6H).

## 4 Discussion

**FIGURE 7:**
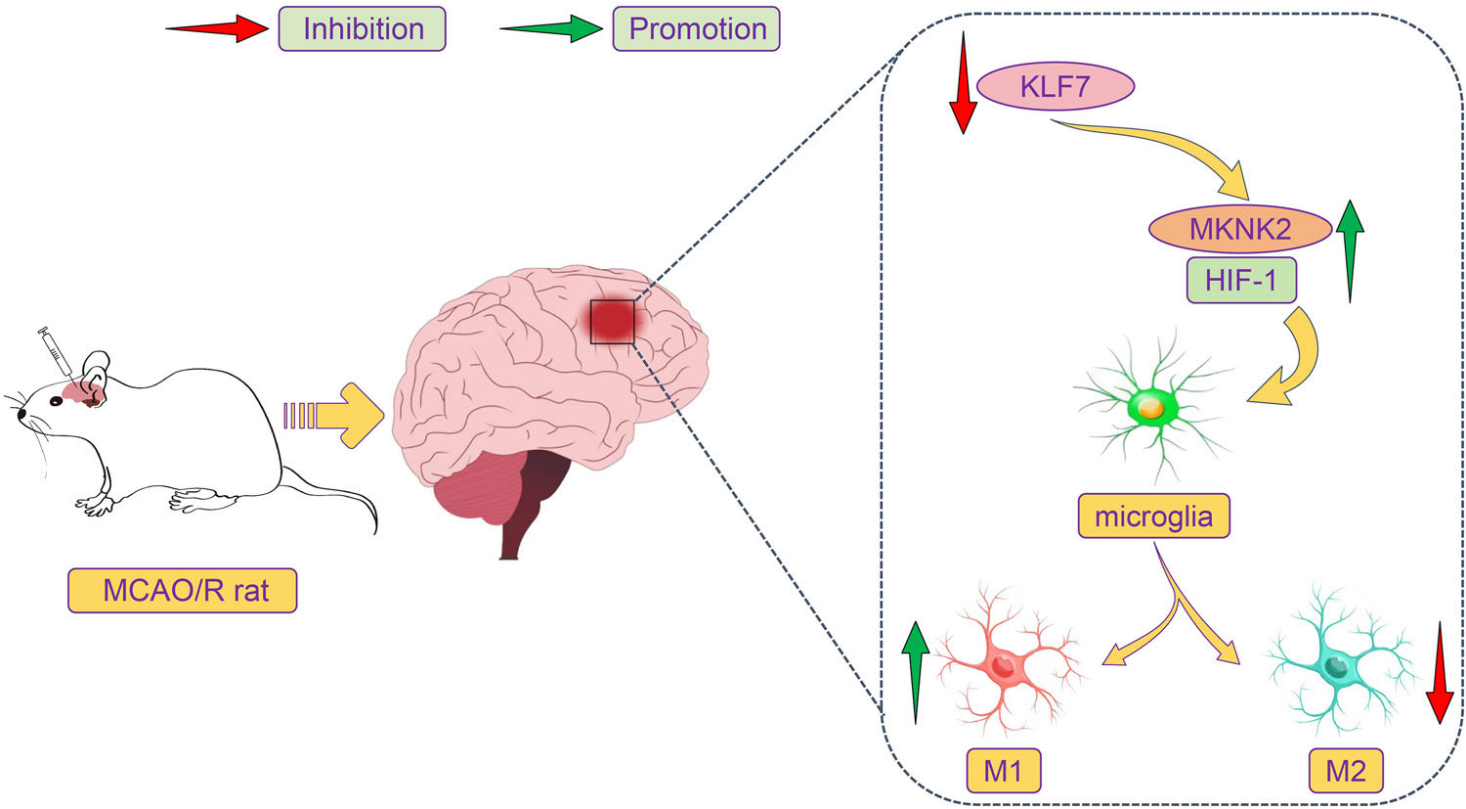
Schematic diagram. Low Krueppel‐like factor 7 (KLF7) expression after IS further impairs transcriptional repression of MAP kinase‐interacting serine/threonine‐protein kinase 2 (MKNK2), which exacerbates middle cerebral artery occlusion/reperfusion (MCAO/R)‐induced pathological injury in rats by activating the hypoxia‐inducible factor‐1 (HIF‐1) pathway‐mediated pro‐inflammatory M1 polarization of microglia.

In the current study, our main findings were as follows: (1) MKNK2 and HIF‐1α were found to be increased in microglia after IS; (2) MKNK2 knockdown alleviated brain injury and inhibited the M1‐like microglia polarization in rats after IS by blocking the HIF‐1 pathway; (3) KLF7 bound to the MKNK2 promoter and further repressed the transcription of MKNK2, thereby alleviating the brain injury and M1‐like microglia polarization in IS. Our findings reveal an important role of the KLF7/MKNK2/HIF‐1 axis in IS, and targeting this axis may be a promising strategy to alleviate IS‐induced brain injury (Figure 7).

The levels of HIF‐1α were found to be increased in rats with cerebral ischemia/reperfusion injury and the cultured microglia following OGD insult (S. F. Chen et al. 2020). The MKNKs phosphorylate eukaryotic initiation factor 4E at serine 209 (X. Jin et al. 2021), which increases the rate of mRNA translation into HIF‐1α protein (Masoud and Li 2015). These insights might explain why MKNK2 was enriched in the HIF‐1 pathway in our bioinformatics prediction. Therefore, we hypothesized it was the overexpression of MKNK2 in microglia that led to the activation of the HIF‐1 pathway in IS. Activated microglia can be classified into “detrimental” M1 versus “protective” M2 subtypes according to their protein/cytokine expression profiles, where M1 microglia (detected by cell surface markers such as CD16 and CD32) secrete pro‐inflammatory cytokines, such as IL‐1β and IL‐6, while M2 microglia (identified by CD206 and Arg‐1) secrete anti‐inflammatory cytokines, such as IL‐10 (Lyu et al. 2021). In the present study, we observed the microglia polarization toward the M2 phenotype in vitro following knockdown of MKNK2. The enhanced expression of the M1 microglia marker and repressed expression of the M2 marker in response to the HIF‐1 activator treatment further corroborated that MKNK2 induced the HIF‐1 pathway activation to promote the pro‐inflammatory response of microglia in IS. Dreas et al. identified indazole‐pyridinone derivatives as a novel class of potent and selective MKNK inhibitors that protect against endotoxin‐induced septic shock by reducing the levels of pro‐inflammatory cytokine TNF‐α and IL‐6 in serum (Dreas et al. 2021). These preclinical findings not only validate MKNK as a driver of systemic inflammation but also highlight the translational potential of MKNK inhibitors. Given the central role of excessive cytokine release in both septic shock and IS, the advancement of these compounds, or their next‐generation analogs, into early‐phase clinical trials could offer a novel anti‐inflammatory strategy to limit neurovascular injury, reduce infarct size, and improve neurological recovery in stroke patients.

It has been suggested that IS may be effectively treated by modulating microglia polarization through transcription factors (Jiang et al. 2020). KLF7 was identified as a core transcription factor in the injury‐ and regeneration‐associated module following peripheral nerve injury (Zhang et al. 2022). The transcription repressor role of KLF7 has been verified in osteoporosis (C. Chen et al. 2022) and possibly in axon degenerative events (Kreymerman et al. 2019). Here, we identified KLF7 as an upstream transcription factor of MKNK2. KLF11 knockout mice exhibited significantly larger brain infarction and poorer neurological outcomes in response to ischemic insults by directly regulating IL‐6 in the brains of ischemic mice (Tang et al. 2018). Uric acid treatment reduced infarct volume and Evans blue leakage by improving KLF2 expression in spontaneously hypertensive rats (Vila et al. 2019). As for the role of KLF7, it has been reported to elicit protection for retinal ganglion cells and descending propriospinal neurons after retinal ischemia‐reperfusion injury (Z. Li et al. 2021) and spinal cord injury (W. Y. Li et al. 2017). More relevantly, KLF7 reduced ipsilateral hippocampal atrophy, decreased the injured cortex volume, and increased the number of 5‐bromo‐2′‐deoxyuridine‐positive neurons by enhancing the phosphorylation of JAK2 and STAT3 in the ipsilateral hippocampus after traumatic brain injury (W. Y. Li et al. 2022). However, its regulatory role in microglia‐mediated inflammatory response has not been validated. Here, the alleviating effects of KLF7 overexpression on IS in rats were found to be overturned by MKNK2 overexpression, further indicating that the MKNK2‐mediated HIF‐1 signaling is the downstream effector of KLF7.

Three major limitations exist in this study. First, for the correlation between elevated total serum LDH with worse functional outcomes and higher recurrence risk in acute IS patients (X. X. Jin et al. 2022), further functional experiments in vitro and in vivo are required to determine whether LDH modulation offers a new avenue for neuroprotection in IS. Second, astrocytic KLF4 inhibited the activation of A1 astrocytes but promoted A2 astrocyte polarization after OGD/R (Wang and Li 2023). Therefore, the role of KLFs in IS might be target‐dependent. Lastly, the knockdown constructs lack cell‐type specificity, and utilizing adeno‐associated virus vectors that achieve specific and efficient gene delivery to microglia (Aoki et al. 2025) is imperative for the elucidation of microglial functions.

## 5 Conclusion

Overall, from our nursing‐led investigation, KLF7 attenuates inflammation and protects neurons in MCAO/R rats by shifting microglial polarization via the MKNK2/HIF‐1 axis, highlighting this pathway as a novel target for IS care.

## Author Contributions

**Ran Wei**: conceptualization, methodology, software, investigation, data curation; formal analysis, visualization, validation, writing – review and editing, writing – original draft. **Shuang Li**: data curation, methodology, investigation, formal analysis, visualization, writing – review and editing, validation. **Lei Tang**: funding acquisition, validation, resources, writing – review and editing, project administration, supervision, visualization, formal analysis, data curation.

## Conflicts of Interest

The authors declare no conflicts of interest.

## Peer Review

The peer review history for this article is available at https://publons.com/publon/10.1002/brb3.70850

## Supporting information




**Supporting Figure**: brb370850‐sup‐0001‐Figure S1.docx

**Supplementary Material**: brb370850‐sup‐0002‐SuppMat.xlsx

## Data Availability

The datasets generated and analyzed during the current study are available from the corresponding author upon reasonable request.
