# Supplementary material for: KLF7 Blocks MKNK2/HIF‐1 Pathway‐Mediated M1 Microglia Polarization to Ameliorate Ischemic Stroke‐Induced Neurological Injury
Source: Brain Behav. 2025 Sep 9;15(9):e70850. doi: 10.1002/brb3.70850 (PMC12417964; doi:10.1002/brb3.70850)
Supplement: Supplementary file 1 — Supporting Figure: brb370850‐sup‐0001‐Figure S1.docx [file BRB3-15-e70850-s002.docx]

**Supplementary Figure 1**


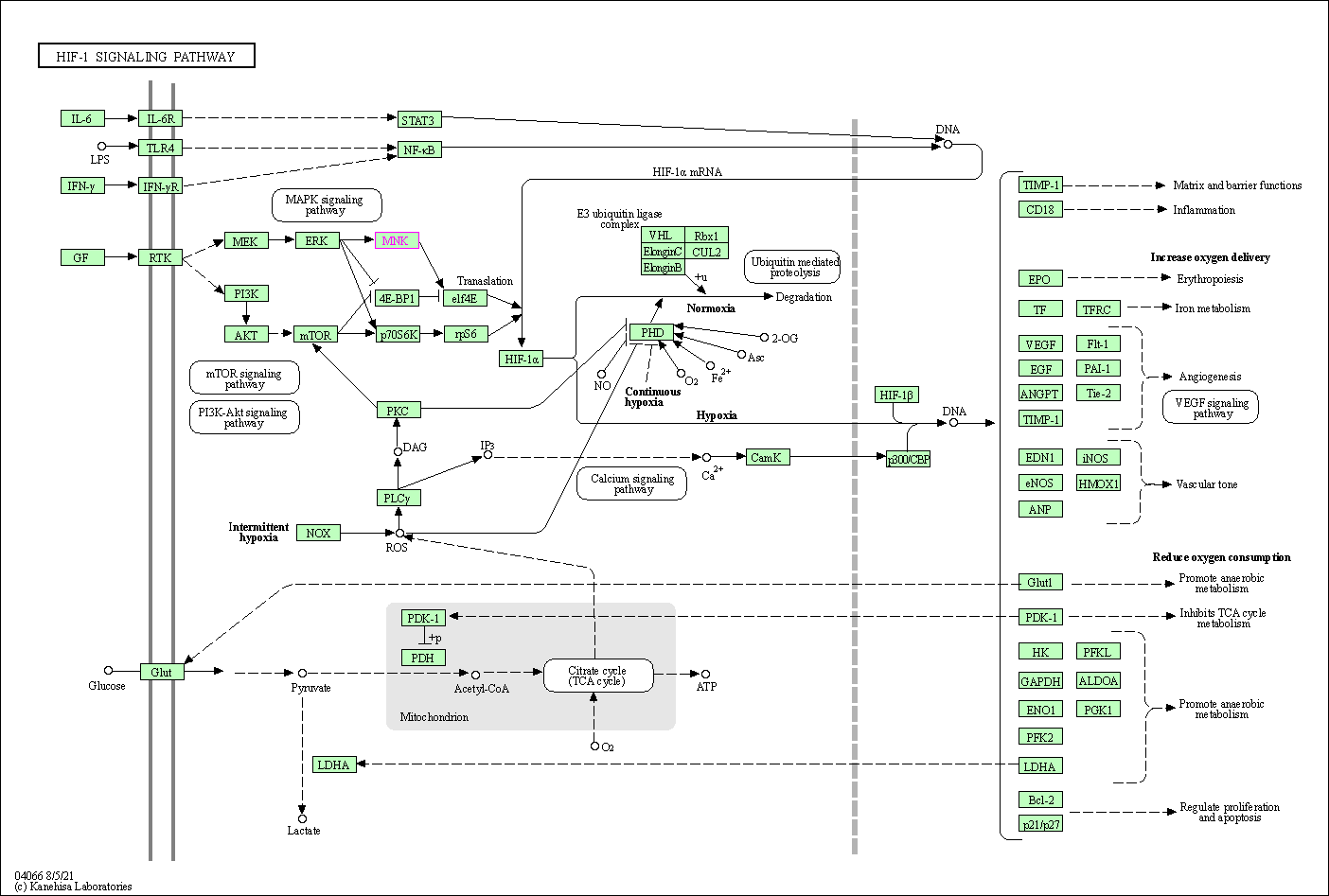


**Supplementary Figure 1** The localization and function of MKNK2 in the HIF-1 pathway.
